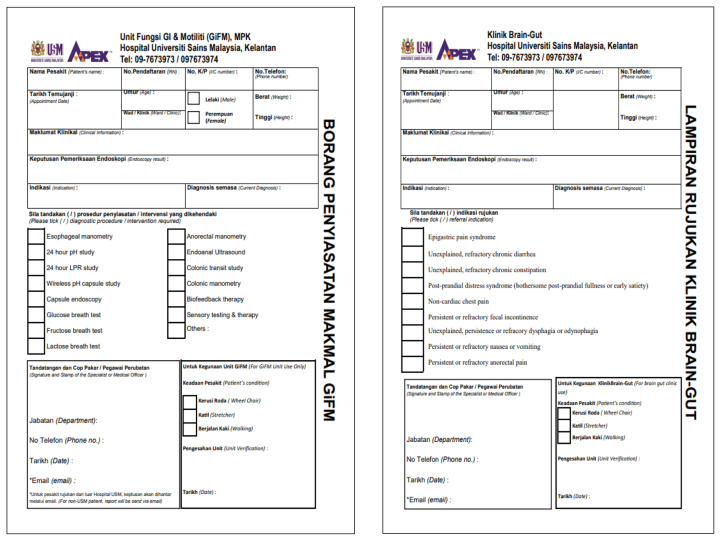# The Brain-Gut Clinic in Hospital Universiti Sains Malaysia: Pioneering New Service to Advance Neuro-Gastroenterology and Motility in Malaysia

**DOI:** 10.21315/mjms2023.30.3.1

**Published:** 2023-06-27

**Authors:** Yeong Yeh Lee, Nabilah Izham, Mohd Faizal Mohd Zulkifly, Mohamed Faiz Mohamed Mustafar, Ahmad Karami Ismail, Nur Farah Fathia Nabila Mohamed Shah, Asrenee Ab Razak, Sanihah Abdul Halim, Zamzuri Idris, Abdul Rahman Izaini Ghani, Muhammad Ihfaz Ismail, Diana Noma Fitzrol, Ang Song Yee, Zaitun Zakaria, Aini Ismafairus Abd Hamid, Nur Asma Sapiai, Norazlina Mat Nawi, Norina Hassan, Jafri Malin Abdullah

**Affiliations:** 1 Department of Internal Medicine, Hospital Universiti Sains Malaysia, Kelantan, Malaysia; 2 GI Function and Motility Unit, Hospital Universiti Sains Malaysia, Kelantan, Malaysia; 3 Brain and Behaviour Cluster, Hospital Universiti Sains Malaysia, Universiti Sains Malaysia, Kelantan, Malaysia; 4 Department of Neuroscience, Hospital Universiti Sains Malaysia, Universiti Sains Malaysia, Kelantan, Malaysia; 5 Nutrition and Dietetics Unit, Hospital Universiti Sains Malaysia, Kelantan Malaysia; 6 Department of Psychiatry, Hospital Universiti Sains Malaysia, Kelantan, Malaysia; 7 Neurology Unit, Department of Internal Medicine, Hospital Universiti Sains Malaysia, Kelantan, Malaysia; 8 Department of Radiology, Hospital Universiti Sains Malaysia, Kelantan, Malaysia; 9 Department of Nuclear Medicine, Radiotherapy and Oncology, Hospital Universiti Sains Malaysia, Kelantan, Malaysia; 10 Neuro Intensive Care Unit, Hospital Universiti Sains Malaysia, Kelantan, Malaysia

**Keywords:** gut mobility, neurogastroenterology, brain-gut, clinical services

## Abstract

Neurogastroenterology and motility is a new but advanced subspecialty within gasteroenterology that cater to difficult, persistent and refractory gut-brain symptoms. Hospital USM has the country’s first and new state-of-the art motility lab that was recently launched on the 25 May 2023, and is covered in nationwide media. Another first is the Brain-Gut Clinic, established on the 16 November 2022. The clinic is a new concept that builds on unique multiple disciplines in relation to the gut-brain axis. It is hoped that there will be more awareness on the existence of neurogastroenterology and motility among doctors and community, and that more research can be forthcoming to reduce the disease burden.

## Historical Perspective

Neurogastroenterology and motility is a relatively new but advanced subspecialty within the larger umbrella of gastroenterology and hepatology. Before 1970s, gastrointestinal (GI) motility was anything but an odd niche for the few academic physiologists and pharmacologists interested in the working of smooth muscle. In the 1970s, new technology started to penetrate the clinical scene including the use of endoscopy. Likewise, to study the physiology of human bowels, multi-lumen catheter connected to a pneumohydraulic pump to transmit pressure changes, had become a pioneering technique producing a number of landmark research. When the industry came in the 1980s with highly successful drugs including loperamide and cisapride for treatment of ‘functional GI disorders (FGIDs)’ that the new specialty got its much needed boost.

During the early years, FGIDs are recognised as disorders of gut motor activity but more recently there is a paradigm shift in the understanding of interactions between the gut and brain i.e. the gut-brain axis that provides a deeper understanding of the disorder. In a global epidemiology study commissioned by the Rome foundation, among 70,000 adults, at least one FGID was diagnosed in 40.3% of internet survey and 20.7% of household survey (1). During the recent fourth iteration of the Rome Diagnostic Criteria, FGIDs have been relabeled as disorders of gut-brain interactions (DGBIs) (2).

## Mechanistic Perspective

There are a number of possible mechanisms that underlie DGBIs but peripheral and central sensitisation (a form of neuroplasticity) are among the most studied (3). With peripheral sensitisation, inflammatory mediators would activate and modulate the gut mucosal receptors including TRPV1, TRPA1 and NAV1.8 which resulted in allodynia or hyperalgesia where painful responses become exaggerated. With central sensitisation, psychological or cognitive dysfunction would result in abnormal signaling downstream in the spinal cord especially through decreased modulation of the descending inhibitory pathway. Due to neuroplastic changes in the gut-brain axis, symptoms in DGBIs can be refractory, persistent and difficult to treat. Such patients may benefit from neuromodulators that allow neurogenesis to occur, and the more receptors (e.g. acetylcholine, dopamine, serotonin and norepinephrine) a neuromodulator can act on, the efficacy is better (e.g. amitriptyline) but adverse events may be more too (4).

Another important mechanism of DGBIs would be dysmotility. For example, in a patient with dysphagia, after exclusion of obstructive causes in the esophagus (including cancer), dysmotility disorders need to be considered. Achalasia is one such disorder and this condition can be diagnosed reliably using the high-resolution esophageal manometry. Another example is a patient with constipation, where defecatory disorder can be a cause and this condition requires evaluation using the high-resolution anorectal manometry. Effective treatment is available in treating certain dysmotility conditions. In the case of achalasia, per-oral endoscopic myotomy or POEM is a recently developed endoscopic technique that has been shown to be beneficial from clinical trials. Likewise, with defecatory disorders, biofeedback therapy is helpful based on a number of clinical trials.

## Causative Perspective

It is equally important to understand what are the triggering factors of neuroplastic changes in the gut-brain axis. There is a growing research interest in gut microbiota being the primary factor. It is found that the gut microbiota interacts closely with the gut immune system and the gut-brain axis (5). Beneficial microbiota or probiotics including lactobacilli and bifidobacterium, produce anti-inflammatory small chain fatty acids (SCFAs) (e.g. butyrate) that can protect the gut against the more harmful bacteria including *Clostridium difficile* and others. These beneficial microbes may be harmed through indiscriminate prescription of antibiotics or through other environmental factors including climate change (6).

Food antigen is the other key triggering factor, and the FODMAPs are probably the most studied in DGBIs (7). FODMAPs is the acronym for fermentable oligosaccharides, disaccharides, monosaccharides and polyols; essentially these are short-chain carbohydrates commonly found in certain fruits and vegetables that are not easily digested or absorbed by the gut (8). As these sugars are readily fermented by the bacteria, the produced gas would stretch the large bowel resulting in symptoms (e.g. bloating). A low FODMAPs diet have been shown in clinical trials to benefit DGBIs (9).

Global warming is fast becoming the biggest global health threat and unpredictable floods are among the many manifestations of climate change (10). We have reported that new onset DGBIs were not unsurprisingly common after a major flood, possibly due to expansion of environmentally derived pathobionts in the bowels of flood victims associated with poor water, sanitation and hygiene practices (11). Furthermore, we have reported beneficial effect of a probiotic in improving mental health of flood victims with DGBIs (12).

## Practice in Malaysia

In Malaysia, DGBIs are perhaps the most common clinical consults in gastroenterology practice and patients are often affected not just by symptoms but by poor quality of life and associated psychological illnesses including anxiety and depression (13). Patients are often investigated with many tests but at the end they may be told to be normal. This often leads to frustrations not just from patients but also doctors. Specialised neurogastroenterology laboratory is extremely scarce in Malaysia and not all centres provide comprehensive testing and or treatment. This is because motility equipments can be expensive and need people who are trained to perform and to analyse the tests.

Hospital Universiti Sains Malaysia (HUSM) in Kota Bharu is probably the only hospital in Malaysia with a highly specialised neurogastroenterology unit, the GI function and motility unit, also known as the ‘my gut brain centre’ (www.mygutbraincenter.com) and this unit is listed under the clinical programmes for DGBI (Appendix A, Gut feelings—the patient’s story) endorsed by the Rome Foundation, an international organisation responsible for the development of Rome Diagnostic Criteria.

The centre caters to motility tests of upper and lower GI tracts, provides specialised treatment programme, research programme and training programme of local and international fellows since 2013. For the past one decade, the centre had performed over 2,000 tests, treated almost 1,500 patients (including referrals outside of Kota Bharu), trained 11 fellows (eight local and three international) and acquired research grants over RM2 million. We have recently acquired more equipments to cater for better services, and more training and research initiatives. In addition, the centre provides specialised consults to other specialties including surgery, paediatric, gynaecology and the dysphagia clinic (a multi-disciplinary clinic).

## The Brain-Gut Clinic

**Figure 1:**
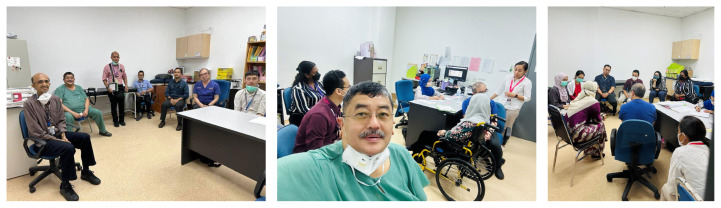
Photographs taken during Brain-Gut Clinic in the months of January and April 2023, with attendance by patients both locally and outside Kota Bharu and experts from clinical neurosciences, gastroenterologists, dietitians and clinical psychologists

**Figure 2:**
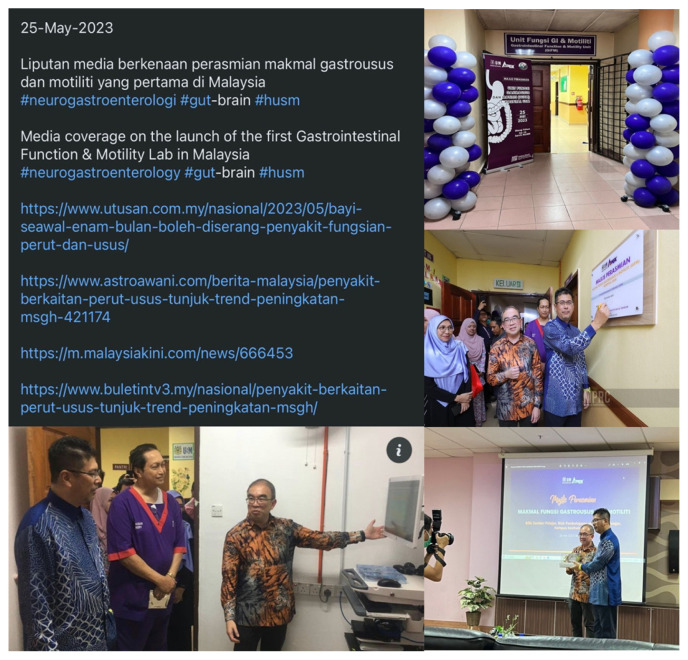
Launching and media coverage of the new state-of-the-art neurogastroenterology and motility lab on the 25 May 2023 by the hospital director, Dato’ Professor Dr. Nik Hisamuddin Nik Ab. Rahman

More recently in 16 November 2022, the Brain-Gut Clinic has been established under the Brain Behavioural Cluster, School of Medical Sciences and HUSM, and this is the first such clinic exists in Malaysia. This clinic is unique in that multiple disciplines are involved included dietary, psychology, psychiatry, neuroscience and of course neurogastroenterology. Patients referred to the clinic were those from all disciplines or specialties with refractory or persistent symptoms of DGBI or dysmotility requiring multi-disciplinary input (proforma shown in Tables 1 and 2). Since its humble operation beginning January 2023, there have been monthly sessions with each session having approximately three patients (Figure 1). As the clinic is a relatively new concept, the referral paths are not matured and need further refinement. Likewise, the multi-disciplinary nature requires time commitment from various experts but also entails new learning experience. Recently, the Gastrointestinal Function and Motility (GiFM) lab was launched on the 25 May 2023 and covered in nationwide media (Figure 2). This is the first and new motility lab in the country, which provide state-of-the-art diagnostic tests and therapy for patients within and outside Malaysia.

## Conclusion and Future Perspective

First, abnormal neuroplasticity of the gut-brain axis and dysmotility may explain refractory symptoms of DGBIs. Second, gut microbiota and food antigens are recognised as important triggers and more recently climate change, too. Third, while the practice of neurogastroenterology and motility is relatively new in Malaysia but it caters to a large disease burden. Fourth, the new Brain-Gut Clinic is a new concept that builds on unique multiple disciplines in relation to the gut-brain axis. From a future perspective, it is hoped that there will be more awareness on the existence of neurogastroenterology and motility among doctors and community, and that more research can be forthcoming to reduce the disease burden.

## Figures and Tables

**Table 1:** A proforma to guide referrals for the Brain-Gut Clinic, Hospital USM, Kota Bharu

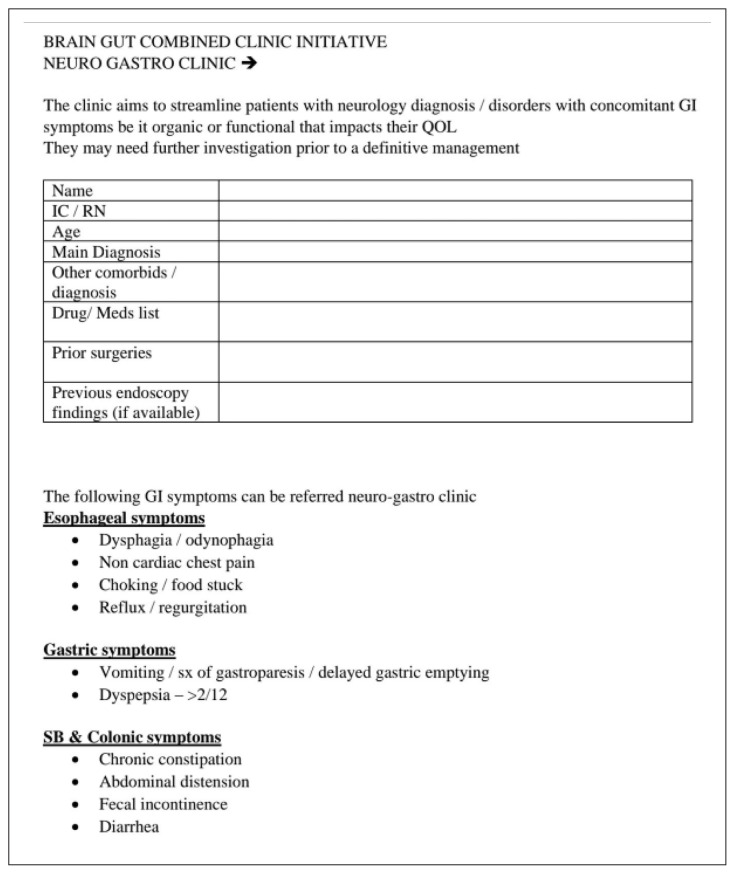

**Table 2:** Request forms by the lab and the clinic for referral purposes